# A Hybrid Rule-Based and Machine Learning System for Arabic Check Courtesy Amount Recognition

**DOI:** 10.3390/s23094260

**Published:** 2023-04-25

**Authors:** Irfan Ahmad

**Affiliations:** 1 Information and Computer Science Department, King Fahd University of Petroleum & Minerals, Dhahran 31261, Saudi Arabia; irfan.ahmad@kfupm.edu.sa; Tel.: +966-013-8601243; 2 SDAIA–KFUPM Joint Research Center for Artificial Intelligence, Dhahran 31261, Saudi Arabia

**Keywords:** Arabic check image processing, courtesy amount recognition, digit recognition, machine learning, pattern recognition

## Abstract

Courtesy amount recognition from bank checks is an important application of pattern recognition. Although much progress has been made on isolated digit recognition for Indian digits, there is no work reported in the literature on courtesy amount recognition for Arabic checks using Indian digits. Arabic check courtesy amount recognition comes with its own unique challenges that are not seen in isolated digit recognition tasks and, accordingly, need specific approaches to deal with them. This paper presents an end-to-end system for courtesy amount recognition starting from check images as input to recognizing amounts as a sequence of digits. The system is a hybrid system, combining rule-based modules as well as machine learning modules. For the amount recognition system, both segmentation-based and segmentation-free approaches were investigated and compared. We evaluated our system on the CENPARMI dataset of real bank checks in Arabic. We achieve 67.4% accuracy at the amount level and 87.15% accuracy at the digit level on the test set consisting of 626 check images. The results are presented with detailed analysis, and some possible future work is identified. This work can be used as a baseline to benchmark future research in Arabic check courtesy amount recognition.

## 1. Introduction

Automatic check processing is an important area of research in the field of pattern recognition. Paper checks, although declining in popularity due to the proliferation of digital payment systems, are still widely used around the world [1]. More than 10 billion checks circulated in 2020 (cf. [2]). Many checks are still processed manually by humans across the world. Automatic processing of checks can save a significant amount of time and cost.

A check has many information fields that need to be filled and subsequently processed. The transaction amount is written as numerals and is termed the *courtesy* amount. Additionally, the amount is also written in words and is known as *literal* or *legal* amount. The courtesy amount and the legal amount must match. A check also has a date field, a check number, and a field to enter the account number. A check also has a signature field to be filled by the payee or on his or her behalf. Much progress has already been made in automatic check processing from checks written in different languages and countries, such as English (e.g., [3] ), French (e.g., [4,5]), Swiss (e.g., [6]), Chinese (e.g., [7,8,9]), Brazilian (e.g., [10,11]), and Indian (e.g., [12,13]), and there are many commercial products already available for automated check processing (e.g., A2iA, Mitek, Parascript, and SoftPro, cf. [14]); however, automatic processing of Arabic checks is still an ongoing research problem. This is partly due to the limited availability of resources, including the lack of public datasets for research in Arabic check processing. Moreover, Arabic check processing has its own challenges due to the peculiarities of the language as well as the local and regional norms and standards of writing checks.

**Figure 1:**
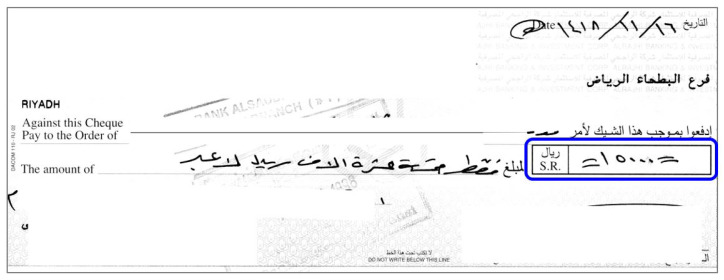
A sample Arabic check image with the courtesy amount highlighted by a blue box.

**Figure 2:**
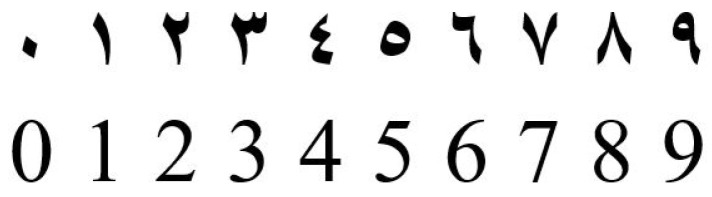
Indian digits from 0 to 9 are shown at the top and the corresponding Arabic digits at the bottom.

Recognizing the courtesy amount from the check image is an important step in automatic check processing. Figure 1 shows a sample Arabic check image with the courtesy amount highlighted. In the Arabic writing system, Indian digits are used for numbers. It is also important to note that although Arabic is written from right to left, numbers are written from left to right. Figure 2 shows the 10 Indian digits alongside the same 10 Arabic digits used in the Roman writing system.

**Figure 3:**
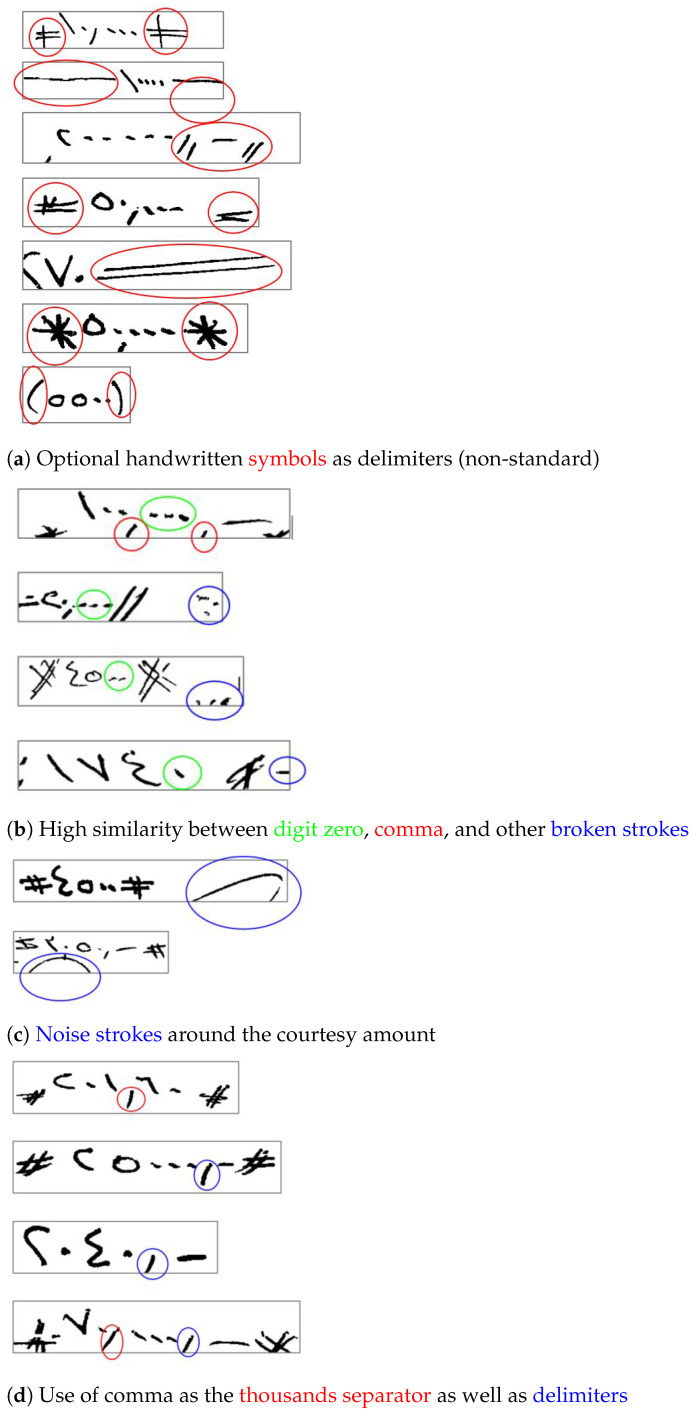
An illustration of some of the peculiar challenges related to Arabic check courtesy amount recognition.

The elementary aspect of research in courtesy amount recognition is to recognize the numeral string, also known as handwritten digit string recognition (HDSR) (e.g., [15]). There are a total of ten possible digits, in addition to special symbols such as delimiters and number separators. Although recognizing isolated digits may be a relatively easier problem with published works reporting accuracies above 99% (e.g., [16,17]), recognizing the complete courtesy amount is a difficult problem because of a number of reasons apart from the common reasons associated with any handwriting recognition tasks, such as variability in pen-strokes and writing style. In the case of courtesy amount, writers optionally add some special delimiter symbols at the beginning and end of the amounts. There are no fixed rules on which symbols can be used as delimiters, their shapes and sizes. In Arabic checks, the comma symbol has a very high resemblance to the Indian digit *zero*. Additionally, noise and broken strokes can look visually similar to a *zero* and can be easily mistaken to be a part of the amount. The presence of extra strokes in the courtesy amount region due to the presence of segments from signatures can pose some additional difficulties. Moreover, the comma symbol is used, at times, as an amount delimiter in addition to its use as a thousands separator, which can further complicate the task. Figure 3 shows some sample courtesy amounts having one or more of the issues discussed above.

To the best of our knowledge, this is the first work on Arabic check courtesy amount recognition. There are many papers in the literature that address digit recognition from courtesy amounts, but do not address complete courtesy amount recognition. We present a complete pipeline for courtesy amount recognition starting from check images to recognizing the amount as a digit sequence. Moreover, we investigated and compared both segmentation-based and segmentation-free approaches to recognize courtesy amounts. Last but not the least, we evaluate our system on a dataset of real bank check images instead of checks generated in laboratories, which may not capture all aspects of the real-world problem.

The rest of the paper is organized as follows: In Section 2, we present the related work on Arabic bank check processing and courtesy amount recognition. In Section 3, the proposed system for Arabic courtesy amount recognition is presented. In Section 4, we present the experimental details, the results, and discussions. Finally, in Section 5, the conclusion and some possible future work are presented.

## 2. Related Work

Automatic check processing involves several important modules, such as courtesy amount recognition (e.g., [18,19,20]), literal amount recognition (e.g., [21,22,23,24,25]), date verification (e.g., [26,27]), and signature verification (e.g., [13,28]) in addition to tasks such as recognizing check numbers and account numbers. Interested readers can refer to [29] for an old but relevant survey on Arabic check processing and to [14] for automatic check processing, in general. The CENPARMI dataset of Arabic bank checks [30] is the only available research dataset that was prepared using real bank checks. As mentioned before, no published work on automatic recognition of courtesy amounts from real Arabic checks has been reported in the published literature to the best knowledge of the author. Researchers have reported courtesy amount digit recognition using presegmented digits available as part of the CENPARMI dataset. Accordingly, in this section, we briefly present the major works related to courtesy amount digit recognition using the CENPARMI dataset of presegmented digits from courtesy amounts. A summary table including the evaluation scores for the reviewed works are presented in Section 4.2.

Sadri et al. [31] presented one of the earliest works on digit recognition from Arabic checks. The authors presented a new feature extraction technique that computes features from all four sides of the size-normalized digit images. A total of 16 features are extracted from each side. The features are based on measuring the distance between the side of the image and the start of the handwritten stroke. The extracted features are used with a support vector machine (SVM) classifier with a radial basis function (RBF) kernel for digit recognition. The one-vs-all setup was used with SVM for the multiclass classification problem. Akbari et al. [32] presented a dataset of Persian bank checks that is made available on request. The dataset consists of two sets, one containing 500 check images to assist research in check image analysis and recognition. The other set consists of 200 real bank checks in addition to 200 forged checks and 100 forms to assist researchers in signature verification tasks.

Juan and Vidal [33] presented digit recognition from binarized digit images using Bernoulli mixture models. Each digit class is represented by a mixture model from a Bernoulli distribution. The model is trained using the maximum likelihood estimation (MLE) technique. The digit images are preprocessed for size normalization before estimating the model parameters. Cheriet et al. [34] investigated the use of 2-layer neural networks and SVMs for digit classification. Several statistical features, such as freeman directions, stroke curvatures, and morphological features, such as mountain, valley, and hole regions, are extracted from the digit images. For the neural network system, the authors used *tanh* activations in the hidden layer consisting of 272 neurons. For the SVM system, the authors used the one-vs-one setup, thereby training a total of 45 classifiers. Both polynomial and RBF kernels were investigated. The SVM-based system gave the best overall recognition rate.

Alamri et al. [35] presented the use of gradient features with SVM classifiers for digit recognition. In addition, the authors presented a rule-based technique for separating digits from the numeral string. A number of prepossessing steps were performed on the images, such as image binarization, size normalization, and noise removal, before extracting the features. Gradient magnitude and direction are computed per pixel using diagonal directions. The output directions are quantized into 32 levels such that each bin represents an interval of $\frac{\pi }{16}$. A Gaussian filter is used to downsample the output before normalizing the features. The RBF kernel was used for the SVM classifier. In addition, the authors employed the one-vs-one training strategy.

Mahmoud and Al-Khatib [36] presented the use of log-Gabor filters for extracting features from digit images. The filtered image was segmented into patches of 3×3, and the mean and variance values from each patch were used as features. A combination of different scale and orientation values was investigated. A number of classifiers were used for evaluating the performance, including SVMs, K nearest neighbor (KNN), and hidden Markov models (HMMs). The best results were achieved using the SVM classifier with four scales and six orientations. An earlier version of the techniques with spatial Gabor features was presented by Mahmoud [37].

Gimenez et al. [38] presented a discriminative version of the Bernoulli mixture model that was trained using maximum mutual information (MMI) criteria as opposed to the commonly used MLE criteria. A log-linear model—a mixture of multiclass logistic regression—was proposed and evaluated on the digit recognition task. Awaida and Mahmoud [39] presented digit recognition using HMM and SVM classifiers. The images are binarized and divided into patches such that the ink pixel distribution is uniform across all the patches. The authors extracted three types of features—gradient, structural features, and concavity—from the digit images. The SVM classifier with the RBF kernel gave better recognition results as compared to the HMM classifier. Assayony and Mahmoud [40] presented a feature learning approach based on the bag-of-features (BoF) framework for digit recognition. Local scale invariant feature transform (SIFT) features are used along with the BoF framework. Several enhancements were performed to suit the framework for Arabic handwriting recognition tasks, such as the use of dense sampling instead of the commonly used Harris detector and the use of soft assignment instead of hard assignment during clustering for codebook generation.

Ahmad [41] presented isolated digit recognition using statistical features computed from local pen geometries. The digit images were segmented into equal width stripes and nine features were computed from each strip. Five different classifiers—SVM, random forest, KNN, naive Bayes, and multilayer neural networks—were investigated for the task. The SVM classifier gave the best results. However, the KNN classifier was the best performing classifier in the presence of a high number of mislabeling in the training set.

Based on the literature review, we can see that many works have been published on handwritten digit recognition for Arabic bank checks, but no work has been reported on complete courtesy amount recognition using real bank check images.

## 3. A System for Arabic Check Courtesy Amount Recognition

In this section, we present a complete system for Arabic check courtesy amount recognition. The system is a hybrid rule-based and machine learning system where some modules are rule-based while the core recognition system employs machine learning. The decision to use a hybrid approach was motivated by considering the positive aspects of each technique. Rule-based techniques can be effectively employed in situations where little or no training data are available and have high precision if used in specialized domain-specific tasks. Machine learning techniques avoid the need for handcrafted rules and can generalize well if a sufficient amount of training data are available. In our case, some modules of the system are very domain-specific, and we do not have enough data to train those modules. Accordingly, we use rule-based techniques for such modules. On the other hand, the core recognition module is generic in nature, and we have sufficient data to train that module robustly. The use of hybrid rule-based and machine learning systems is a common practice in the area of pattern recognition (e.g., [42,43,44]), including document processing (e.g., [21]). For the courtesy amount recognition module, we propose two different approaches, one segmentation-based and one segmentation-free approach and compare their performances. Investigating segmentation-free approaches using deep learning-based sequence models can be an interesting future work. While such methods have the potential to be effective, a key challenge in their application is the limited availability of large datasets of annotated Arabic bank checks.

**Figure 4:**
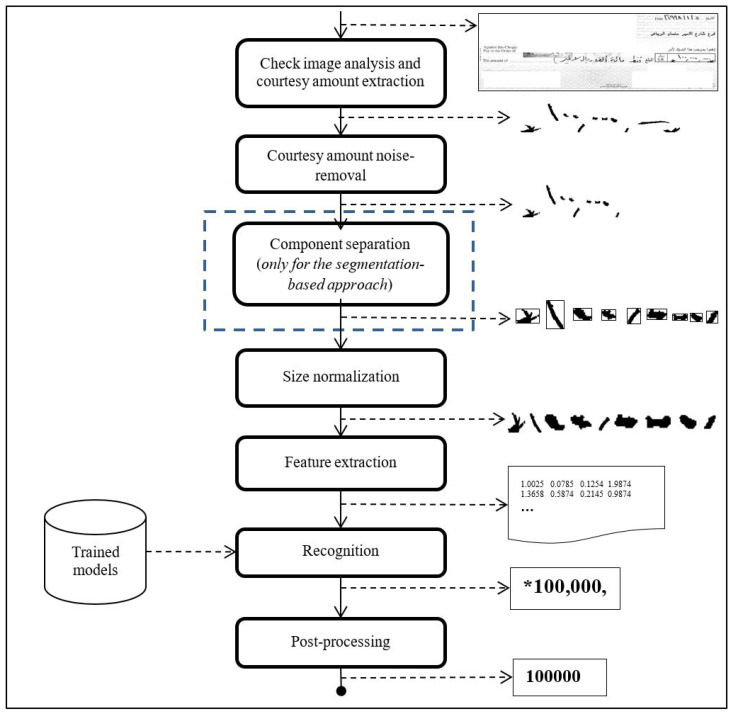
An illustration of the courtesy amount recognition system.

Figure 4 illustrates our system for courtesy amount recognition from Arabic check images. The complete check image is input to the system, as illustrated in the top part of the figure. The first step is to extract the courtesy amount region from the check image. A rule-based system is employed for this task. The extracted region is fed to the preprocessing module, which constitutes several steps, including the removal of unwanted components from the extracted region. This is followed by a segmentation step, where the components of the courtesy amount are separated. The segmentation step is only applied for the segmentation-based approach. The segmented components are finally size normalized. Features are extracted from each image component in the next stage to be fed to a machine learning-based handwritten digit recognition system. The digit recognition system is trained on isolated digits and symbols, such as the comma (,) and dot (.), and boundary strokes typically used by writers on Arabic checks, such as the star (*) and hash (#). In the case of the segmentation-free approach, features are extracted from the extracted courtesy amount images using a sliding window approach and a machine learning model is trained to recognize the courtesy amount. The output of the recognition system is postprocessed to remove the symbols from the digit sequence. The postprocessed digit sequence is the final output from the system representing the courtesy amount. The details of the major system components are presented next.

### 3.1. Check Image Analysis and Courtesy Amount Extraction

This is the first major module of the system. A scanned check image is input to the system and fed to this module to extract the courtesy amount region from the complete check image. A rule-based top-down approach for document image segmentation is employed for the task, as it is an effective approach where the document layout is known beforehand. The module was adapted from the check analysis and component extraction technique presented in [45].

The check image is skew corrected as a first step. The technique is based on connected component analysis and pixel projections. As a check image has a number of printed horizontal lines on it, detecting the skew of these lines can help in correcting the skew of the check image. The horizontal lines can be efficiently detected by the pixel projection technique after removing smaller components from the check image. Dynamic thresholding is employed to identify the smaller components in the image based on computing the average size of the components in the image. Once the pixels from individual horizontal lines are identified, regression is performed to estimate the slope of each horizontal line. The skew angle of the check image is computed by averaging the slopes of all the detected horizontal lines. Finally, the skew is corrected by rotating the image in the opposite direction of the skew angle.

The region of interest is extracted from the skew-corrected image as a next step. First, smaller components are removed from the check image using connected component analysis. The courtesy amount region is located in the check image using the pixel projection technique. A rectangle region is finally cropped from the check image to extract the courtesy amount region. The skew correction and courtesy amount extraction techniques are summarized in Algorithm 1.
**Algorithm 1** Skew Correction and Courtesy Amount Extraction**Input**: scanned check image**Output**: courtesy amount region   1:**procedure** SkewCorrection                ▹ skew correction of check image2:    1. Binarize the check image3:    2. Remove small components from the check image using connected-component analysis4:    3. Identify printed horizontal lines using pixel projections5:    4. Estimate the slope of each horizontal line by performing regression on the pixels of the line6:    5. Compute skew of the check image by averaging all the estimated slopes7:    6. Correct the skew by rotating the image in the opposite direction of the skew8:**end procedure**  9:**procedure** CourtesyAmountExtraction ▹ courtesy amount extraction from check image10:    1. Binarize the check image11:    2. Remove small components from the check image using connected-component analysis12:    3. Locate the courtesy amount region using pixel projections13:    4. Extract the courtesy amount region by cropping the check image14:**end procedure**

### 3.2. Preprocessing Courtesy Amount

The extracted courtesy amounts undergo a number of preprocessing steps before features can be extracted from them. As a first step, noise removal is applied to eliminate unwanted strokes and scanning noise from around the courtesy amount. In Arabic checks, writers occasionally use long strokes at the beginning and end of the amount as delimiters. The noise removal step also attempts to remove such strokes, if present. This is performed using connected component analysis. It should be noted here that a conservative approach, as opposed to an aggressive approach, is applied. Aggressive removal risks losing useful components. On the other hand, if unwanted components are still present, most of them can still be dealt with by the later steps of the current module or even by later modules such as the recognition system or the postprocessing module.

The noise-removed image is then segmented into individual components. As mentioned before, this step is employed only for the segmentation-based approach to recognize courtesy amounts. This step is straightforward and is based on identifying all the connected components in the courtesy amount image. It should be noted that further filtering is carried out at this step to remove unwanted components. Some heuristics based on the height-to-width ratio are employed to filter out the components that are deemed nondigits with high probability. The parameters of the heuristic are set by applying the process to the training set images, independent of the test set. The segmented and filtered components are finally size normalized by resizing all the images to the same height. In the case of the segmentation-free approach, the noise-removed courtesy amount image (as opposed to the segmented components) is height normalized. The aspect ratio of height and width is maintained for each image. The preprocessing module is summarized in Algorithm 2.
**Algorithm 2** Preprocessing Courtesy Amount**Input**: courtesy amount image**Output**: size-normalized image(s)   1:**procedure** NoiseRemoval    ▹ remove unwanted noise from around the written amount2:    1. Remove small components from the periphery of the image using connected-component analysis3:    2. Identify and remove long horizontal or vertical strokes4:**end procedure**  5:**procedure** ComponentSeparation▹ extract individual components from the image6:    1. Extract connected components from the noise-removed courtesy amount image7:         1.1. Record the relative position of each connected component8:    2. Filter out a connected component if:9:         2.1. The height-to-width ratio of the component is beyond a threshold, AND10:       2.2. The connected component is at the periphery of the amount region11:**end procedure**  12:**procedure** SizeNormalization         ▹ height normalize the images13:    1. Normalize the height of each image to $h_{norm}$14:         1.1. Record the current height (h) and width (w) of each image15:         1.2. The new height of each image is $h_{norm}$16:         1.3. New width of each image $=\frac{h}{w}\times h_{norm}$17:**end procedure**

### 3.3. Feature Extraction

**Figure 5:**
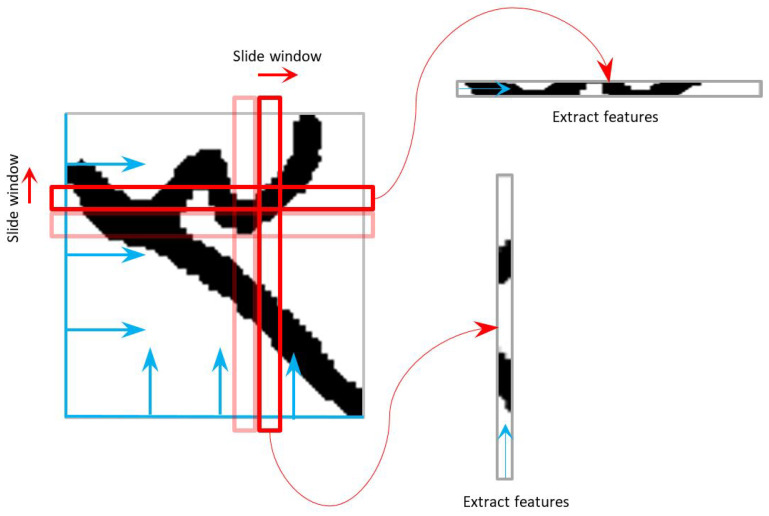
An illustration of the feature extraction process from two sides of the normalized images. All the extracted features are concatenated at the end.

Once the images are normalized, features are extracted from them to be fed to a classifier for training. We extract statistical features from local pen-stroke geometries on the image. Our approach is adapted from [46]. We use a sliding window approach where a window of a predefined width having the same height as the image is slid over an image from one end to the other. Features from the image slice under a window are extracted and concatenated as a feature vector. For the segmentation-based approach, we perform this process twice, once sliding the window from left to right and another time from top to bottom. Figure 5 illustrates the feature extraction approach. For the segmentation-free approach, the process is performed only once by sliding the window from left to right across the extracted courtesy amount (as compared to segmented digits in the segmentation-based approach).

Nine features are extracted from each image slice. Three of these features are related to the local stroke contours—the average distance of the lower contour to the base of the image, the average distance of the upper contour to the base of the image, and the average distance of the center-of-gravity of the stroke pixels to the base of the image. Three features are related to the orientation of the strokes—the orientation of the lower contour, the orientation of the upper contour, and the orientation of the average distribution of the stroke pixels. The orientations are computed by performing linear regression on the pixel positions of the pixels selected. In addition, we compute the average of the number of stroke to nonstroke transitions in each column of an image slice. The average of the distance between the top-most and the bottom-most stroke pixels for each column of an image slice is used as the eight feature. Finally, the ratio of the number of stroke pixels to the total number of pixels in an image slice is used as the ninth feature. The sliding window technique and the feature extraction process is summarized in Algorithm 3.
**Algorithm 3** Sliding Window and Feature Extraction**Input**: size-normalized images, width *W* of the sliding window**Output**: extracted features from the images   1:**procedure** SlidingWindow    ▹ move a window of predefined width across the image2:    1. Place the window of width *W* and height $h_{norm}$ at the start of the image3:    **while** window not at the end of the image **do**4:        2.1. *imageSlice* = Crop the image slice under the sliding window;5:        2.2. features = ExtractFeatures(imageSlice)6:        2.3. $features_{all}=\left[features_{all};features\right]$  ▹ combine feature vectors from the individual image slices to a global feature matrix7:        2.4. Shift the sliding window by *W* pixels8:    **end while**9:    3. return the extracted features $features_{all}$10:**end procedure**  11:**procedure** ExtractFeatures(imageSlice) ▹ extract statistical features from the local geometry of the image slice12:    1. Extract the following features from the *imageSlice*:13:         $d_{l}$ = average distance of the lower contour from the image base14:         $d_{u}$ = average distance of the upper contour from the image base15:         $d_{c}$ = average distance of the center-of-gravity of ink pixels from the image base16:         $s_{l}$ = slope of the lower contour17:         $s_{u}$ = slope of the upper contour18:         $s_{c}$ = slope of the center-of-gravity of the ink-pixel contour19:         $n_{t}$ = average of the number of transitions between ink pixel and background per column20:         $p_{d}$ = pixel density of *imageSlice*21:         $a_{d}$ = average of the distance between the bottom-most and the top-most ink pixels in a column22:    2. $features=[d_{l},d_{u},d_{c},s_{l},s_{u},n_{t},p_{d},a_{d}]$ ▹ concatenate individual features into a feature vector23:**end procedure**

### 3.4. Classifier Training and Courtesy Amount Recognition

This is the core machine learning module of our system. The features extracted from the previous module are fed to this module to recognize the courtesy amount. The recognition system first needs to be trained on the different digit classes in addition to a few other symbols, such as delimiters and the comma “,”. For the segmentation-based approach, we trained the system using isolated digit and symbol images so that each class could be clearly differentiated. The training module of the system performs image size normalization and feature extraction prior to training a classifier. The image size normalization and feature extraction follow the same procedure as detailed in the previous sections. We selected *random forest* as the classifier for the recognition system. Random forest was used for the recognition system because it is an ensemble model that is relatively robust to overfitting and generally achieves high recognition accuracy. Moreover, the system is not very sensitive to the hyperparameters of the model, and thus is easy to set up and calibrate. Last but not the least, the training is fast compared to other powerful classifiers such as SVMs.

The random forest is an example of ensemble learning and was introduced by Ho [47] and extended by Breiman [48]. The base learners for random forest are the *decision tree* classifier. The final classification output of the random forest classifier is a majority vote over the output of individual decision trees. A decision tree classifier is a powerful classifier but is prone to overfitting. For a random forest classifier to have high prediction performance, individual trees should have low correlation. To achieve this, individual decision trees are trained on a *bagged* training set. Moreover, only a small subset of features are randomly considered during each node splitting operation while constructing the decision trees. Creating an ensemble of decision trees this way helps in limiting the overfitting, which leads to an overall low bias and low variance classifier (cf. [49], pp. 587–596).

The *number of base learners*, i.e., the number of decision trees, is the most important hyperparameter of the random forest classifier. Another hyperparameter is the number of features to be considered during node-splitting. It is commonly set to $\sqrt{d}$ for classification problems, where *d* is the total number of features. Apart from the hyperparameters of the random forest classifier, the decision tree classifier has its own set of hyperparameters. Important ones are the *maximum depth* of a decision tree and the *minimum number of samples in a node* to continue node splitting.

Our segmentation-free courtesy amount recognition system is based on continuous Hidden Markov Models (HMMs) implemented using the HTK tools [50]. The models were first initialized using the uniform initialization procedure followed by a number of iterations of Baum–Welch training. The trained system was then used to perform forced alignment on the training data. Next, the information from forced alignment was used to initialize individual HMMs (modeling the digits and various symbols) using Viterbi initialization. This was followed by a number of iterations of Baum–Welch retraining. Finally, the trained system was used to decode the evaluation set using the Viterbi algorithm. The feature extraction and the HMM system closely follow the system presented in [51].

### 3.5. Postprocessing

**Figure 6:**
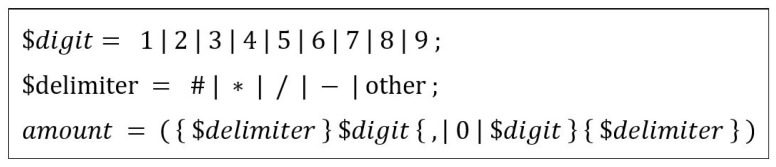
Task grammar for the postprocessing parser.

We are interested in retrieving the courtesy amount as a sequence of digits only. The output from the recognition module is a sequence of symbols that can include digits, optional delimiter symbols at the beginning or end of the amount, and the comma “,”. We parse the sequence output by the recognition system to strip the nondigit symbols as a postprocessing step. It should be noted that leading 0s are also removed as they do not contribute to the amount. Zeroes at the beginning may also possibly be noise components or broken strokes from delimiter symbols or other digits. In Figure 6, we present a simple grammar specified in the extended Backus–Naur form (BNF) form to implement the postprocessing parser.

## 4. Experiments, Results, and Discussion

In this section, we present the details of the experiment we conducted, the results obtained, and some discussions on the performance of our system based on the analysis of the results. We first introduce the dataset used for the experiments.

### 4.1. Dataset

**Figure 7:**
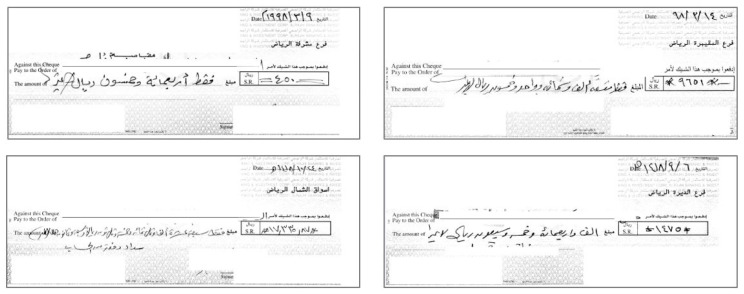
Sample check images from the CENPARMI Arabic check dataset [30].

We used the CENPARMI dataset of Arabic checks to conduct the experiments [30]. This dataset was developed by the Center for Pattern Recognition and Machine Intelligence (CENPARMI), Concordia University. To the best of our knowledge, the CENPARMI dataset is the only available dataset of real bank checks in Arabic. It was collected in collaboration with AlRajhi Banking and Investment Corp., Riyadh, Saudi Arabia. The dataset is not available freely. There is a nominal license fees to use the dataset for research. Sample check images from the dataset are shown in Figure 7.

The dataset contains 2499 annotated check images divided into training and test sets. In addition, specific regions of interest from the check image, such as legal amount and courtesy amount, are made available separately. Furthermore, isolated digit images from courtesy amount and subword images from legal amount are also provided as part of the dataset. This enables researchers to focus on specific research problems based on their interests. We use the annotated check images from the test set to evaluate our courtesy amount recognition system. In Table 1, we present key statistics related to the courtesy amounts from the check images used in our system evaluation. In order to train the classifier, we used the dataset of isolated digits and symbols provided in the dataset. The training set consists of a total of 10,536 images, including 7390 digit images, and the rest of the images belong to symbols such as “,” and delimiters. Digit “0” is the most frequent class in the training set with 3793 samples; whereas, digit “9” with 194 samples is the least frequent class. We would like to mention that the isolated digit images available in the training set are nonoverlapping and independent from the check images available in the test set for system evaluation.

### 4.2. Isolated Digit Recognition

Before evaluating our complete courtesy amount recognition system, we evaluated the performance of our digit classifier. We trained the digit classifier on the isolated digit images of the CENPARMI dataset, which consists of a total of 10,536 images in the training set, as mentioned in the previous section. The test set consists of a total of 3035 digit samples. The distribution of samples across the different classes in the test set is similar to the training set, with digit “0” being the most frequent with 1574 samples and digit “9” being the least frequent with 74 samples. Values for key hyperparameters for the random forest classifier are presented in Table 2. The hyperparameter combination was selected based on the results of 10-fold cross validation on the training set.

**Figure 8:**
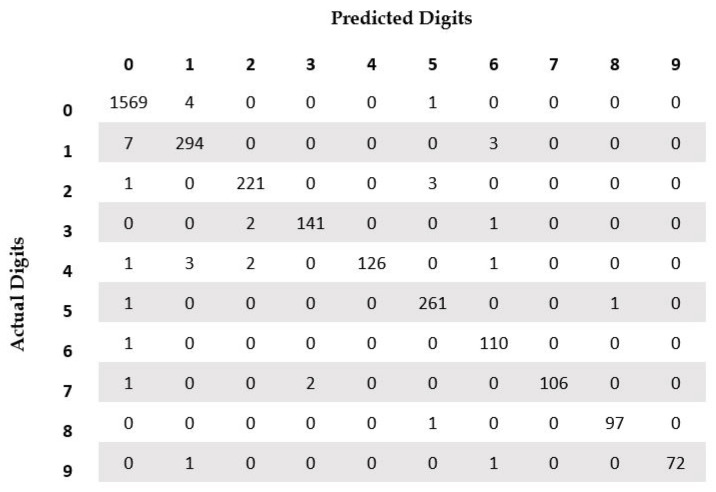
Confusion matrix for isolated digit recognition on the CENPARMI Arabic check digit dataset.

We achieved an overall accuracy of 98.75% on the test set of the isolated digit dataset. In Figure 8, we present the confusion matrix of the results on the test set. From the figure, we can notice that digit “1” is the most misclassified digit, with a total of 10 misclassifications, out of which it was misclassified as digit “0” seven times, and three times as digit “6”. The next most misclassified is digit “4”, with seven misclassifications. Three of the samples were misclassified as digit “1”. Other misclassifications are to digit “0”, “2”, and “6”. Samples for digits “6” and “8” have one misclassification each. Based on the results, we can state that our recognition system is quite robust with high classification accuracy. In Table 3, we present a comparison of our digit recognition system with other published works on the same dataset. We can see from the table that our recognition system has among the best accuracies reported in the literature. Three of the nine published works report higher accuracy compared to ours, but the difference in accuracy is statistically significant for only one among those three works. The difference of two proportions as presented in [52] is used to compute the statistical significance of the results at the 95% confidence level.

### 4.3. Segmentation-Based Courtesy Amount Recognition

In this section, we present the performance of our complete system for courtesy amount recognition. As mentioned previously, we used 626 check images from the test set to evaluate our courtesy amount recognition system. Each check image is input to the system. The process starts with check analysis and extracting the courtesy amount from the check image. Next, the courtesy amount is segmented into individual components, removing some leading and trailing noise strokes from around the amount region. The extracted components are size normalized, and features are extracted from them. The extracted features are used by the classifier to predict the class of each component, which can include optional symbols in addition to the digits. Finally, the postprocessor parses the recognized sequence and outputs the final courtesy amount. In Table 4, we summarize the performance of our courtesy amount recognition system. We can see from the table that we were able to process 422 out of the 626 check images completely free of any mistakes, thereby achieving an end-to-end recognition rate of 67.4%, with a statistical significance of ±3.15%. Additionally, only 1 error was made on 125 out of the remaining 204 check images. Accordingly, 79 check images out of 626 had 2 or more mistakes, representing 12.6% of the samples. Therefore, we claim that the results are very promising, even if further improvement may be needed for commercial viability of the system. Additionally, the results can serve as a benchmark for future research on Arabic courtesy amount recognition, as there are no previously published results on this task on real bank check images.

To understand the performance of the system in more detail, we present the results at the digit level. As some digits might mistakenly be removed during the check analysis and processing stages or extra digits might be added erroneously due to oversegmentation issues, we need to update the accuracy measure to account for such deletion and insertion errors. The accuracy rate is thus defined as (cf. [53], pp. 419–421):

(1) $$Accuracy\left(\%\right)=\left(100-\frac{S+I+D}{N}\right)\times 100$$

where *S*, *I*, and *D* are substitution, insertion, and deletion errors, respectively, and *N* is the total number of digits across all the courtesy amounts in the test set.

**Figure 9:**
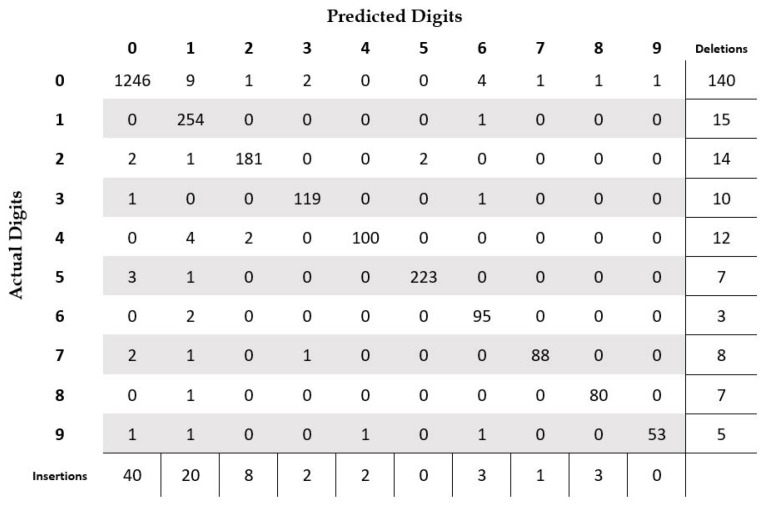
Confusion matrix at the digit level for courtesy amount recognition on the CENPARMI Arabic check courtesy amount dataset (segmentation-based approach).

In Table 5, we summarize the performance of our system at the digit level. We achieved an accuracy of 87.15% with a statistical significance of ±1.1% at the 95% confidence level. The errors due to deletion are the largest source of errors, with a total of 221 deletions. The substitution errors are the smallest concern, with a total of 48 cases. It should be noted here that the deletion and insertion errors are primarily a result of segmentation errors, and not due to the recognition system. The deletion errors are mainly the result of undersegmentation or due to misidentifying digits as noise or delimiter symbols. Similarly, the insertions errors are mainly due to oversegmentation or due to incorrectly identifying noise and delimiters as digits. These issues are not faced in isolated digit recognition tasks as the provided images are already segmented at the digit level. In Figure 9, we present the confusion matrix for different digit classes in addition to the count of insertions and deletions per digit. From the figure, we can clearly see that the majority of deletions are related to digit “0”, which is quite understandable. The digit “0” is a very small glyph and thus might be removed during check image analysis or preprocessing steps. Moreover, it can easily be confused with the comma symbol or considered broken strokes due to check scanning issues. Additionally, for similar reasons, digit “0” has the highest number of insertion errors. However, digit “1” is no longer the most misclassified class, as was the case for isolated digit recognition. In fact, it has only one substitution error. On the other hand, digit “0” is the most misclassified digit, with a total of 19 substitutions. To summarize, we can say that handling digit “0” is one of the most important challenges for the Arabic check courtesy amount recognition task. It should be noted that we did not present the recognition results on the delimiters as the goal of the courtesy amount recognition system is to correctly recognize the amount i.e., the digit string, and the performance on delimiter recognition is not directly relevant to the problem.

### 4.4. Result Analysis

**Figure 10:**
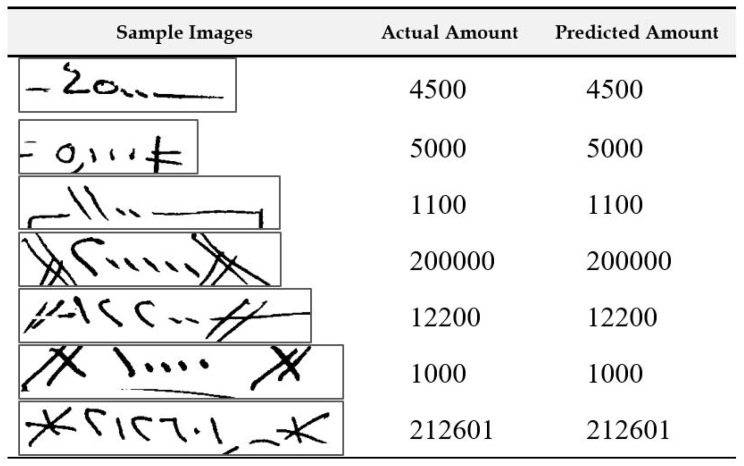
Illustration of samples with no recognition error in the final output of the system.

In this subsection, we present some additional analysis of the results. In Figure 10, we show samples of extracted courtesy amount regions from the check images where the recognition system made no mistakes. The second column shows the actual amount written on the image, and the last column shows the output of the system. We can see from the samples that our recognition system was robust enough to recognize the amounts under varied writing conditions.

**Figure 11:**
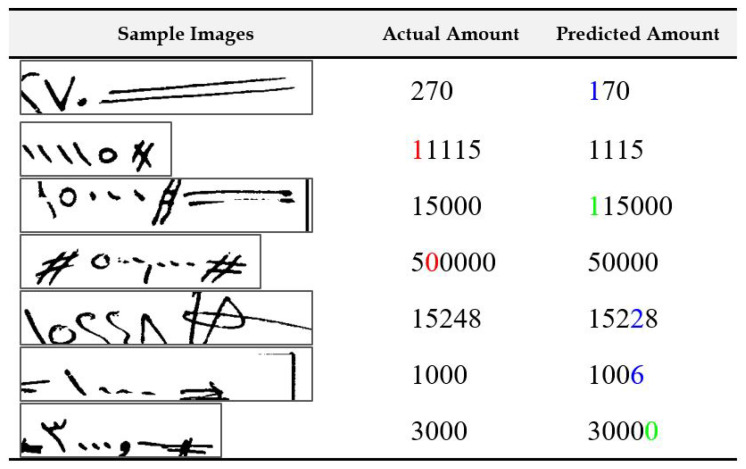
Illustration of samples with a single recognition error in the final output of the system. Note: digits colored red are indications of deletion errors, digits colored green are indications of insertion errors, and digits colored blue are indications of substitution errors.

**Figure 12:**
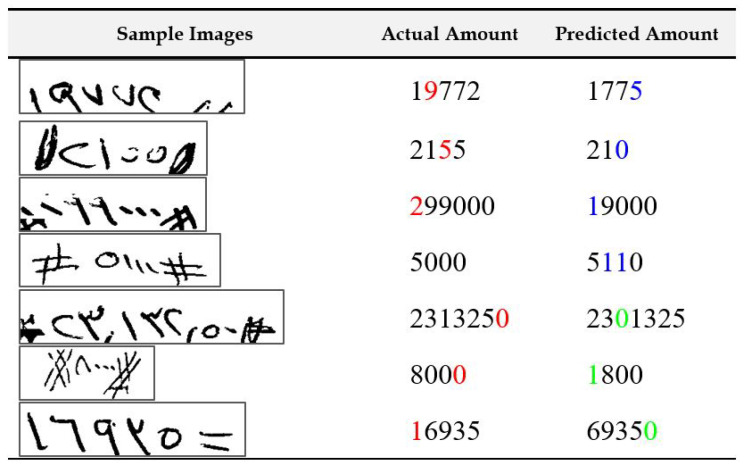
Illustration of samples with with two recognition errors in the final output of the system. Note: digits colored red are indications of deletion errors, digits colored green are indications of insertion errors, and digits colored blue are indications of substitution errors.

In Figure 11, we show samples of extracted courtesy amount regions from check images where the recognition system made only one mistake. The digits colored red in the second column indicate that they were mistakenly deleted by the recognition system. Additionally, digits colored green in the last column indicate insertion errors—digits added mistakenly by the recognition system. The digits colored cyan in the last column indicate substitution errors where the recognition system confused a digit with some other digit. We can see that in the topmost case, digit “2” was substituted as digit “1”. This seems mainly due to the problem of broken digit as a part of digit “2” was written outside the amount box by the writer. The check samples provided in the dataset are grayscale images. Having colored scans, instead of grayscale images, can help in such cases, whereby following the color of the pen-stroke can help recover written portions outside the amount box. In the second example, the first digit “1” was mistakenly deleted by the system. On visual inspection, it is clear that the digit is very short and might have been confused as a noise stroke. It is almost a third of the height of the fourth digit “1” in the sequence. Using context such as from literal amounts can help in such cases. In the third example, the situation is the opposite, as one extra digit “1” is added by the system. We can see that the digit “1” was broken, which led to the system recognizing it as two digits. Digit “0” in the fourth example is deleted by the recognition system, probably because it is considered noise. Improved scanning processes and better preprocessing, such as utilizing image enhancement techniques, may help mitigate such problems. Digit “4” in the next example is recognized as digit “2” as a part of it was lost during the extraction process. The leftover part visually resembles the digit “2”. A digit “0” is mistaken as digit “6” in the next example for no clear reasons. One explanation could be that it was deleted by the system, but the vertical printed border toward the end of the image was mistaken as a “6”. Finally, a “,” seems to be mistaken as a digit “0” in the last example. In Figure 12, we present samples where the recognition system made two mistakes in each amount recognition task. The causes for errors seem similar to the ones explained before. However, it is worth noting that most cases involve at least one deletion error.

**Figure 13:**
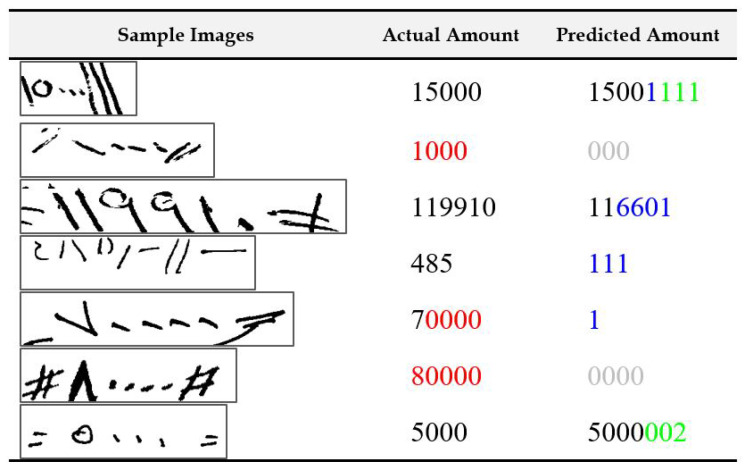
Illustration of samples with three or more recognition errors in the final output of the system. Note: digits colored red are indications of deletion errors, digits colored green are indications of insertion errors, and digits colored blue are indications of substitution errors.

In Figure 13, we present samples where three or more errors were made by the recognition system. There are a few interesting points to note from the samples shown. The most common reasons for multiple mistakes have to do with deletion errors, mainly for digit “0”. In some cases, the amount contains a single nonzero digit in the start followed by a sequence of 0s. If the first digit is deleted by mistake by the recognition system and the remaining 0s are recognized by the system, the postprocessing parser will still delete them as leading 0s are not significant in an amount. These digits are grayed-out in the figure. Using information from literal amounts may help address such issues. Moreover, multiple errors occur mostly together in the sequence, and many times, the type of error is the same in all the instances. This indicates that one root cause of error, such as a broken stroke or a noisy scanning, leads to a sequence of mistakes. Thus, if we can fix that one problem, we can make significant improvements. In general, the recognition system can possibly be improved by using external knowledge such as information from the literal amount and can verify and, if needed, perform further processing if the two amounts do not concur.

### 4.5. Segmentation-Free Courtesy Amount Recognition

We also experimented with a segmentation-free approach to courtesy amount recognition, where we did not segment the courtesy amount into isolated digits after extracting and preprocessing the courtesy amount regions. A sliding window approach to feature extraction was employed similar to that presented in Section 3.3 with the exception that the sliding window moves across the complete preprocessed courtesy amount instead of the segmented regions. Moreover, the sliding window is only moved horizontally from left to right. The width of the window was selected as 4 pixels, and the consecutive windows overlap by 2 pixels such that the window is shifted by 2 pixels (i.e., 4−2=2 pixels) every step we progress to the right. The same nine features listed in Algorithm 3 are computed from the image strip under the sliding window. In addition, we appended nine derivative features for every window frame. Thus, the dimension of the feature vector is 18.

As mentioned in Section 3.4, our segmentation-free amount recognition system is based on continuous Hidden Markov Models (HMMs) implemented using the HTK tools [50]. The system hyperparameters, such as the sliding window width and overlap, the number of states per HMM, and the number of mixtures per state were optimally configured based on the recognition results on the validation set, which consisted of 200 courtesy amounts randomly selected from the training set, independent of the test set. The important hyperparameters of the system are listed in Table 6.

In Table 7, we summarize the performance of our segmentation-free courtesy amount recognition system. We can see from the table that the system was able to process 333 out of the 626 check images free of any mistakes, thereby achieving an end-to-end recognition rate of 53.2%. Additionally, only 1 error was made on 179 out of the 626 check images. Accordingly, 114 check images out of 626 had 2 or more mistakes, representing 18.2% of the samples. Comparing the results from Table 4, we can see that the segmentation-based system clearly outperforms the segmentation-free system at the amount level.

In Table 8, we summarize the performance of our system at the digit level. We achieved an accuracy of 82.61% with a statistical significance of ±1.23% at the 95% confidence level. The errors due to insertion are the largest source of errors, with a total of 191 insertions. The substitution errors are the lowest with a total of 134 cases. In the segmentation-based approach, the insertion and substitution errors are significantly lower as compared to the segmentation-free approach as can be see from Table 5. This can partly be attributed to the power of the random forest classifier. On the other hand, the deletion errors in the segmentation-based approach are higher as compared to the segmentation-free approach. This can be partly attributed to the issue of touching digits, which we are unable to segment effectively.

**Figure 14:**
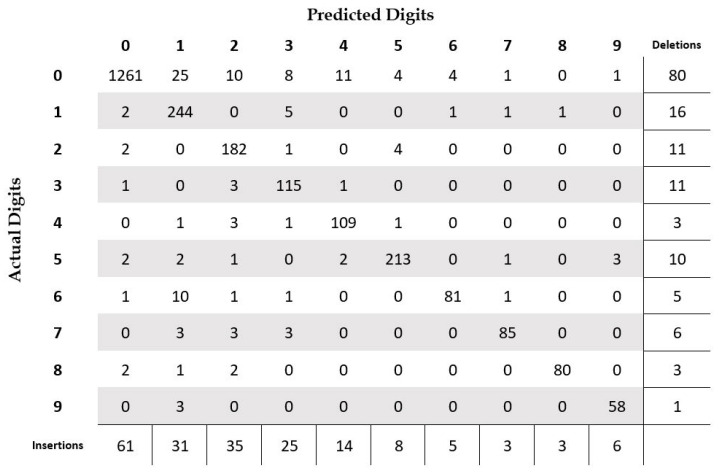
Confusion matrix at the digit level for courtesy amount recognition using the segmentation-free approach on the CENPARMI Arabic check courtesy amount dataset.

In Figure 14, we present the confusion matrix for different digit classes in addition to the count of insertions and deletions per digit. From the figure we can clearly see that, similar to the case of segmentation-based recognition, the majority of deletions and insertions are related to digit “0”. Similarly, digit “0” is the most misclassified digit, with a total of 64 substitutions. However, unlike segmentation-based recognition, the total number of insertion errors are more than the total number of deletion errors. Additionally, the substitution errors are significantly higher for most of the digits when compared to the segmentation-based recognition.

We can clearly see from the results that the segmentation-based approach achieved better overall results than the segmentation-free approach.

## 5. Conclusions and Future Work

Automatic processing of checks is an important area of research in pattern recognition. To date, there is no published work on Arabic check processing using real check images that presents a system that can take check images as an input and recognize the courtesy amount and the literal amount automatically. In this paper, we presented an end-to-end system to automatically process courtesy amounts from Arabic checks. The presented system is a hybrid combining syntactic and statistical pattern recognition approaches. The system processes the check images in stages involving check image analysis and extracting courtesy amount regions from the image. This is followed by the preprocessing stage, where the courtesy amount region is preprocessed and sent to the next stage for feature extraction. A recognition module recognizes the digit sequence along with other symbols from the courtesy amount using the extracted features. Finally, a postprocessing module cleans the recognized amount and outputs the final result from the system. We evaluated the system on the CENPARMI Arabic check dataset. For amount recognition, we presented two alternate systems: a segmentation-based approach using the random forest classifier and a segmentation-free approach using HMMs. The results from both the approaches were compared and analyzed. In-depth analysis of the results was performed to understand the major sources of errors.

As this is the first work to the best of our knowledge on Arabic bank check courtesy amount recognition using real bank checks, there are no other works to compare and benchmark our results with. Another limitation of the work lies in the fact that the dataset is relatively small, with slightly over 2000 samples prepared from only 1 bank. For the presented system, the results achieved are promising, but further improvements are needed to achieve commercial viability. Among the major issues that need improvement is to develop better techniques to deal with digit 0. Segmentation-based recognition showed better performance but the current system still needs improvements in order to deal with touching digits.

We presented a hybrid system in which a rule-based system extracts the components from the courtesy amount for further processing. Investigating segmentation-free approaches using deep learning-based sequence models can be an interesting future work and may prove to be effective for the task; however, one concern with using such systems is the lack of large datasets for Arabic check processing. Using a pretrained deep learning model with transfer learning may be a possible solution to overcome this challenge (e.g., [54]), or to use GAN-based augmentation of training data (e.g., [55]). Automatic processing of Arabic check literal amounts is another interesting future work. The published literature has addressed recognizing presegmented subwords from literal amounts, but no work has yet been reported to process the complete literal amount. Combining the results from courtesy amount and literal amount recognition to make a final prediction on the actual amount may further improve the results and is another possible future work.

## Figures and Tables

**Table 1:** Key statistics related to the courtesy amounts from the test set of the CENPARMI Arabic check dataset.

Total number of samples	626
Average number of digits per sample	4.3
Median number of digits per sample	4
Number of digits in the shortest amount	2
Number of digits in the largest amount	7

**Table 2:** Settings for the important hyperparameters of the random forest classifier.

Number of base learners (decision trees)	200
Number of features randomly selected at node splitting	14
Maximum depth of each decision tree	50
Minimum number of samples for splitting a node	2

**Table 3:** Comparison with other state-of-the-art systems evaluated on the CENPARMI Arabic check isolated digit dataset.

Reference	System Description	Accuracy (%)	Statistical Significance
[31]	SVM with distance features	94.14	±0.74
[33]	Multivariate Bernoulli mixtures	≈98.00	±0.46
[34]	SVM with statistical and structural features	98.18	±0.44
[37]	KNN with spatial Gabor filters	97.99	±0.51
[35]	SVM with gradient features	98.48	±0.41
[36]	SVM with log Gabor features	98.95	±0.35
[38]	Log-linear model based on Bernoulli mixtures	98.00	±0.46
[39]	SVM with gradient, structural, and concavity features	99.04	±0.34
[40]	SVM with SIFT bag-of-features	99.34	±0.29
[41]	SVM with features from local geometries of pen strokes	98.65	±0.40
Present work	Random forest with statistical features on local geometry	98.75	±0.38

**Table 4:** Summary of the results for courtesy amount recognition using the segmentation-based approach on the CENPARMI Arabic check dataset.

Number of samples with no error (percentage)	422 (67.4%)
Number of samples with only one error (percentage)	125 (20%)
Number of samples with two errors (percentage)	38 (6.1%)
Number of samples with three or more errors (percentage)	41 (6.5%)

**Table 5:** Summary of the results at the digit level for courtesy amount recognition on the CENPARMI Arabic check dataset.

Accuracy	87.15%
Total deletion errors (percentage)	221 (8.2%)
Total insertion errors (percentage)	79 (2.9%)
Total substitution errors (percentage)	48 (1.8%)

**Table 6:** Settings for the important hyperparameters of the segmentation-free HMM-based recognition system.

Sliding window width	4 pixels
Sliding window stride	2 pixels
HMM topology	Bakis
Number of states per HMM	14
Number of mixtures per state	12

**Table 7:** Summary of the results for courtesy amount recognition using the segmentation-free approach on the CENPARMI Arabic check dataset.

Number of samples with no error (percentage)	333 (53.2%)
Number of samples with only one error (percentage)	179 (28.6%)
Number of samples with two errors (percentage)	66 (10.5%)
Number of samples with three or more errors (percentage)	48 (7.7%)

**Table 8:** Summary of the results at the digit level for courtesy amount recognition using the segmentation-free approach on the CENPARMI Arabic check dataset.

Accuracy	82.61%
Total deletion errors (percentage)	146 (5.4%)
Total insertion errors (percentage)	191 (7.1%)
Total substitution errors (percentage)	134 (4.9%)

## Data Availability

This paper uses third Party Data. Licensed data were obtained through Centre for Pattern Recognition and Machine Intelligence (CENPARMI), Concordia University, Canada. Please visit the link https://www.concordia.ca/research/cenparmi/resources.html (accessed on 12 February 2023) for more details on obtaining the dataset.
